# Human in vitro modeling of adjuvant formulations demonstrates enhancement of immune responses to SARS-CoV-2 antigen

**DOI:** 10.1038/s41541-023-00759-y

**Published:** 2023-10-26

**Authors:** Simon Doss-Gollin, Sanya Thomas, Byron Brook, Kimia Abedi, Célia Lebas, Floriane Auderset, Yamile Lugo-Rodriguez, Guzman Sanchez-Schmitz, David J. Dowling, Ofer Levy, Simon D. van Haren

**Affiliations:** 1 Precision Vaccines Program, Boston Children’s Hospital, Boston, MA 02115 USA; 2 Harvard Medical School, Boston, MA 02115 USA; 3 Vaccine Formulation Institute, 1228 Plan-les-Ouates, Geneva, Switzerland; 4 Broad Institute of MIT and Harvard, Cambridge, MA 02142 USA

**Keywords:** Adjuvants, Adjuvants

## Abstract

Adjuvants can enhance vaccine immunogenicity, but their mechanism of action is often incompletely understood, hampering rapid applicability for pandemic vaccines. Herein, we characterized the cellular and molecular activity of adjuvant formulations available for pre-clinical evaluation, including several developed for global open access. We applied four complementary human in vitro platforms to assess individual and combined adjuvants in unformulated, oil-in-water, and liposomal delivery platforms. Liposomal co-formulation of MPLA and QS-21 was most potent in promoting dendritic cell maturation, selective production of Th1-polarizing cytokines, and activation of SARS-CoV-2 Spike-specific CD4^+^ and CD8^+^ T cells in a co-culture assay. Select formulations also significantly enhanced Spike antigen-specific humoral immunity in vivo. This study confirms the utility of the cumulative use of human in vitro tools to predict adjuvanticity potential. Thus, human in vitro modeling may advance public health by accelerating the development of affordable and scalable adjuvants for vaccines tailored to vulnerable populations.

## Introduction

As one of the most successful and cost-effective public health interventions, vaccines prevent around five million deaths globally every year^1^. The impact of infectious diseases and resulting damages recently became evident during the COVID-19 pandemic^1–3^. While currently authorized and/or approved SARS-CoV-2 vaccines have demonstrated efficacy and proved lifesaving, their potential is limited by the need for freezing of mRNA formulations, multiple booster dose requirement, and reduced immunogenicity in vulnerable populations such as children, older adults, and immunocompromised individuals. Thus, there remains an urgent unmet need to develop safe and effective vaccines that can be quickly deployed on a global scale in the event of a pandemic or epidemic^4^. Adjuvanted vaccines reduce the number of immunization doses required to induce immune responses, resulting in increased availability for global supply. For example, the formulation of GlaxoSmithKline’s *Fendrix* with AS-04 adjuvant and hepatitis B antigen achieved dose sparing, reducing doses from three to two^5,6^.

Vaccines comprised solely of purified protein antigens often produce little or no T cell response and do not induce the appropriate antibody (Ab) response with a single dose, especially at the extremes of life^7^. The need for multiple immunizations to achieve adequate Ab response is a challenge that can be overcome by formulating a vaccine antigen with adjuvants to enhance the efficacy of weak antigens and induce desired immune responses. An adjuvant is a component of a vaccine that enhances and/or shapes the magnitude, breadth, and/or durability of antigen-specific immune responses, and has been used in vaccine development since late 19th century^8^. Vaccines currently licensed in the US and/or Europe for use in the development of human vaccines include adjuvants such as aluminum salts; oil-in-water emulsions like MF-59, AS-03 and AF-03; virosomes; and AS-04, a monophosphoryl lipid A (MPL) preparation with aluminum salt)^7^. Selection of the adjuvant and its formulation depends on the nature of the antigen used in vaccine development, the desired type of immune response, the age of the target population, and the route of vaccine administration^7^.

A major challenge to the development of low-cost vaccines that would benefit low- and middle-income countries is the restricted access to vaccine- or adjuvant-formulations, in part due to protection by intellectual property laws^9^. While IP protection of adjuvants is key to on-going discovery and development of novel adjuvants, it may also limit adjuvant research and impair global access of vaccines^10^. Therefore, we included in our study the mechanistic evaluation of adjuvant formulations developed for global open access, as well as select other adjuvants currently being evaluated in clinical trials. We evaluated these adjuvants in four distinct but complementary human in vitro platforms: (1) the whole blood assay (WBA), to inform the magnitude of immune activation and specificity towards distinct cell types, and incorporating the unperturbed extracellular soluble factors^11,12^; (2) a microphysiological human tissue construct assay (HTC), to measure the autonomous development and maturation of migratory Antigen-presenting cells^13^; (3) the monocyte-derived dendritic cell assay (MoDC), to assess the ability of adjuvants to promote differentiation of different T-helper subsets^14,15^; and (4) the dendritic cell-T cell interface assay (DTI), to measure the ability of adjuvants to boost the processing and presentation of SARS-CoV-2 Spike protein and activate antigen-specific CD4^+^ and CD8^+^ T cells^16,17^. Our hypothesis was that studying MOA of these adjuvants would inform the optimal dosage and formulation needed to induce T-helper cell type 1-mediated (Th1) immunity and/or CD8-mediated immunity considered favorable for protection against respiratory viral infections. We found that the liposomal co-formulation of MPL and QS-21, which is comparable to the licensed adjuvant system AS-01, stimulated the development of T-helper 1 cells and promoted the induction of both CD4^+^ and CD8^+^ T cells against SARS-CoV-2.

## Results

### LS formulation attenuates reactogenicity potential of MPL in whole-blood assay

**Fig. 1:**
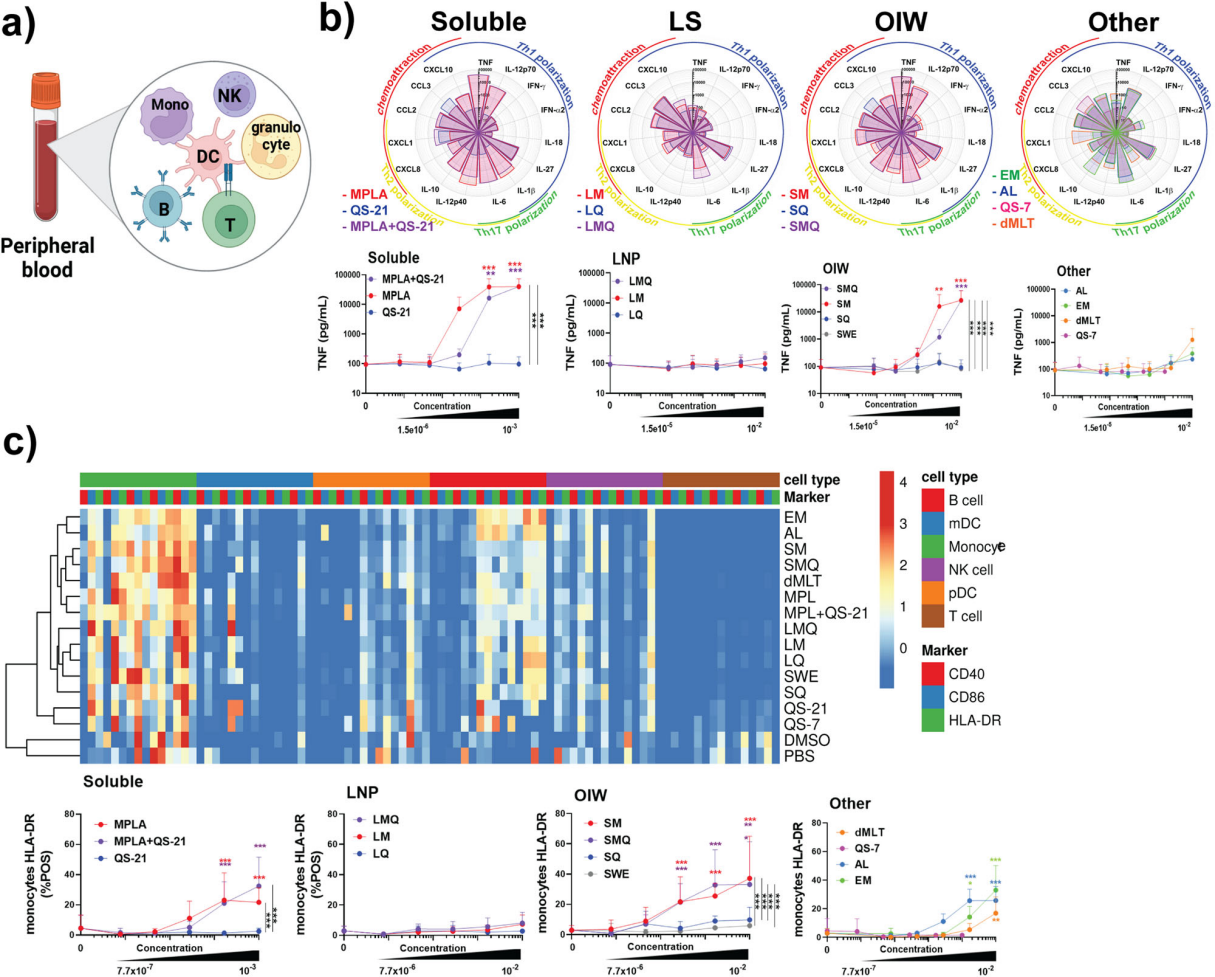
MPL-containing compounds potently induce innate immune response in whole blood assay. **a** Schematic representation of the Whole Blood Assay (WBA). **b** Flower plots show expression levels of 21 cytokines in pg/mL induced by treatment of whole blood with adjuvants at a 1:100 dilution for LNP, OIW, and Other, and 1:1000 dilution for Soluble formulations. Dose-response curves (mean + SD) indicate TNF production across a five-point dilution series, along with a vehicle control. Top concentrations reflect least diluted, as above. Further dilutions were 1/6 (v/v) in RPMI for all formulations. **c** Heat map indicating log10 MFI of activation markers across cell types. Dose-response curves measuring percentage of HLA-DR^+^ monocytes. Stars above a data point on dose-response curves indicate statistical significance relative to vehicle control using a two-way repeated measures ANOVA and Dunnet’s method for multiple comparison testing. Stars adjacent to vertical bars on dose-response curves (mean + SD) indicate statistically significant differences between treatment groups at a given concentration using a two-way repeated measures ANOVA with Tukey’s multiple comparison testing. *N* = 5 for all readouts, except CXCL8 where *N* = 2. (*p < 0.05, **p < 0.01, ***p < 0.001).

The effects of MPL- and QS-21-containing adjuvants, as well as lipidated TLR7/8 agonist 3M-052, formulated either in an oil-in-water (OIW) emulsion (EM) or with Aluminum hydroxide (AL), and double-mutant heat-labile enterotoxin (dmLT), were evaluated in vitro using whole blood from five adult donors (Fig. 1a). MPL and QS-21 were evaluated separately or in combination as these have been reported to act synergistically in vivo^18^, but not extensively tested in vitro and are also used together in licensed vaccine formulations^19,20^. To assess the effect of formulation, these adjuvants were also tested in OIW and in liposomes (LS). Whole blood samples were stimulated across a five-point dose-response curve of each formulation tested and the levels of 21 cytokines in the supernatant were quantified. Optimal concentration ranges were determined by assessing cytokine induction versus cytotoxicity across a wide range of concentrations (Fig. 1b, Supplementary Fig. 2). Soluble formulations and OIW emulsions of MPL and QS-21 produced significantly increased levels of TNF, IL-12p70, IFNγ, IL-6, IL-10, CXCL1, CCL2, and CXCL10 as well as decreased levels of CCL3 relative to vehicle controls. Soluble MPL and QS-21 also induced the production of IL-12p40 and IL-1β, while OIW emulsions induced IL-18 production. This broadly immunogenic profile was comparable to that produced by the 3M-052-based adjuvants EM and AL as well as to dmLT. In contrast, LS formulations were more selective in inducing cytokines, only increasing production of IL-12p70, IL-6, CXCL8, CCL2, and CXCL10. Among MPL- and QS-21-containing adjuvants, MPL was the primary driver of activation, with adjuvants containing QS-21 in the absence of MPL inducing increased expression of only IL-12p70, CCL2, and CXCL8 (Fig. 1a). The MPL-driven effect and the impact of formulation type can be clearly observed by TNF for which no increase in expression was seen relative to vehicle controls when whole blood was treated with QS-21-containing adjuvants or LS formulations, while soluble formulations or OIW emulsions which contained MPL resulted in upregulation of TNF expression (Fig. 1b).

To identify specific cell types that were activated by the formulations, we measured cell surface markers using flow cytometry. Cells were characterized as B cells (CD20^+^), mDCs (CD1c^+^), monocytes (CD14^+^), NK cells (CD56^+^), plasmacytoid DCs (CD123^+^), or T cells (CD3^+^) per Supplementary Fig. 3. Activation was quantified based on expression of CD40, CD86, and HLA-DR. We found that most adjuvant formulations primarily activated monocytes, with some activation of pDCs and NK cells (Fig. 1c). In addition, the 3M-052-containing formulations EM and AL induced higher levels of B cell activation, which is in accordance with TLR7 expression in these cells. Among monocytes, activation was mainly seen in MPL-containing formulations and was generally lower in LS formulations.

### LS formulations drive monocyte differentiation to a mature dendritic cell-like fate in human tissue construct assay

**Fig. 2:**
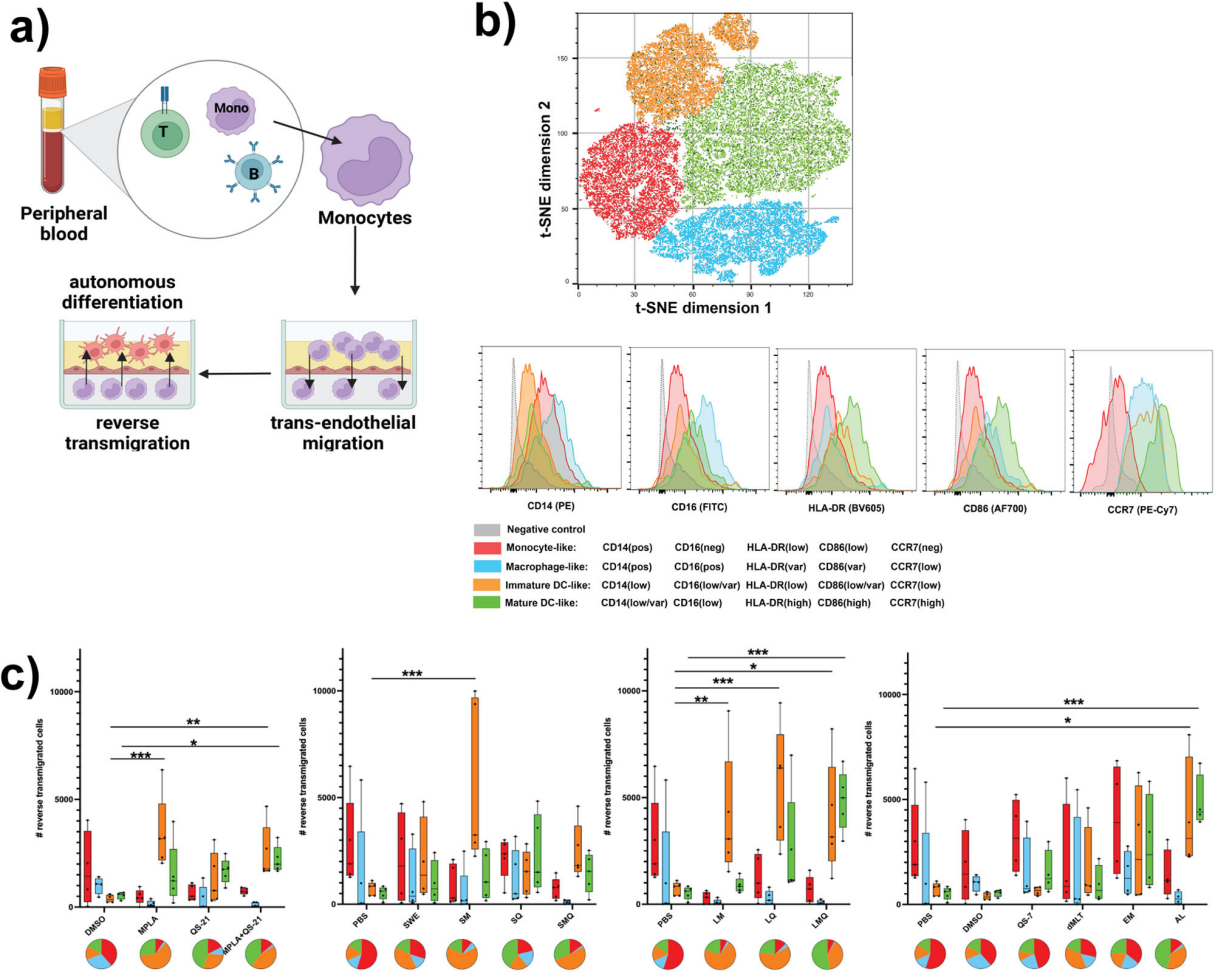
LS-containing formulations drive monocytes to a DC-like fate in human tissue construct assay. Monocytes were stimulated inside the tissue construct with adjuvants at a 1:100 dilution for LNP, OIW, and Other, and 1:1000 dilution for Soluble formulations, before flow cytometric analysis of treatment-induced migrated cells. **a** Schematic representation of the Human Tissue Construct (HTC) assay. **b** Dimensional reduction by t-distributed stochastic neighbor embedding identifies distinct clusters of phenotypically similar reverse-transmigrated cells. Differential expression of surface markers among clustered populations demonstrates similarity to phenotypes of known cell types. **c** Box plots indicate reverse-transmigrated cell counts separated by cell phenotype for each treatment condition at its highest concentration (centre line: median; bounds: 25th/75th percentile; whiskers; lowest/highest point). Pie charts for each condition show the fraction of each phenotype within the total population of reverse-transmigrated cells. Stars indicate a statistically significant difference in reverse-transmigrated cells of a given phenotype relative to the vehicle using an ordinary two-way ANOVA with Dunnet’s method for multiple comparisons. *N* = 5 for all assays. (*p < 0.05, **p < 0.01, ***p < 0.001).

To characterize the ability of MPL and QS-21-containing adjuvants to influence cell fate decisions in a microphysiological setting, we utilized a human tissue construct model of monocyte extravasation and in-tissue autonomous differentiation as previously described^13^. CD33-expressing monocytes were cultured in autologous plasma on top of a confluent monolayer of endothelial cells grown over collagen cushions. After stimulating these cells for 48 h with adjuvant formulations, we harvested the reverse-transmigrated cells and characterized them by flow cytometry (Fig. 2a). Using an unbiased gating strategy utilizing t-distributed stochastic neighbor embedding (t-SNE), we identified four distinct phenotypic clusters among the reverse-transmigrated cells (Fig. 2b). Based on this approach, we characterized these populations as monocyte-like (CD14pos, CD16neg, HLA-DRlow, CD86low, CCR7neg), macrophage-like (CD14pos, CD16pos, HLA-DRvar, CD86var, CCR7low), immature DC-like (CD14low, CD16low/var, HLA-DRlow, CD86low/var, CCR7low), or mature DC-like (CD14low/var, CD16low, HLA-DRhigh, CD86high, CCR7high).

As expected in this model, in the absence of adjuvant stimulation, reverse-transmigrated cells were primarily monocyte-like or macrophage-like. In contrast, in all but one case, stimulation with formulations containing MPL produced a significant increase in immature DC-like phenotype (Fig. 2c). Furthermore, among both soluble and LS-based formulations, combining MPL and QS-21 also significantly increased the number of reverse-transmigrated cells with a mature DC-like phenotype.

### LS-based formulations induce a robust Th1-polarizing cytokine response in human MoDCs in the MoDC assay

**Fig. 3:**
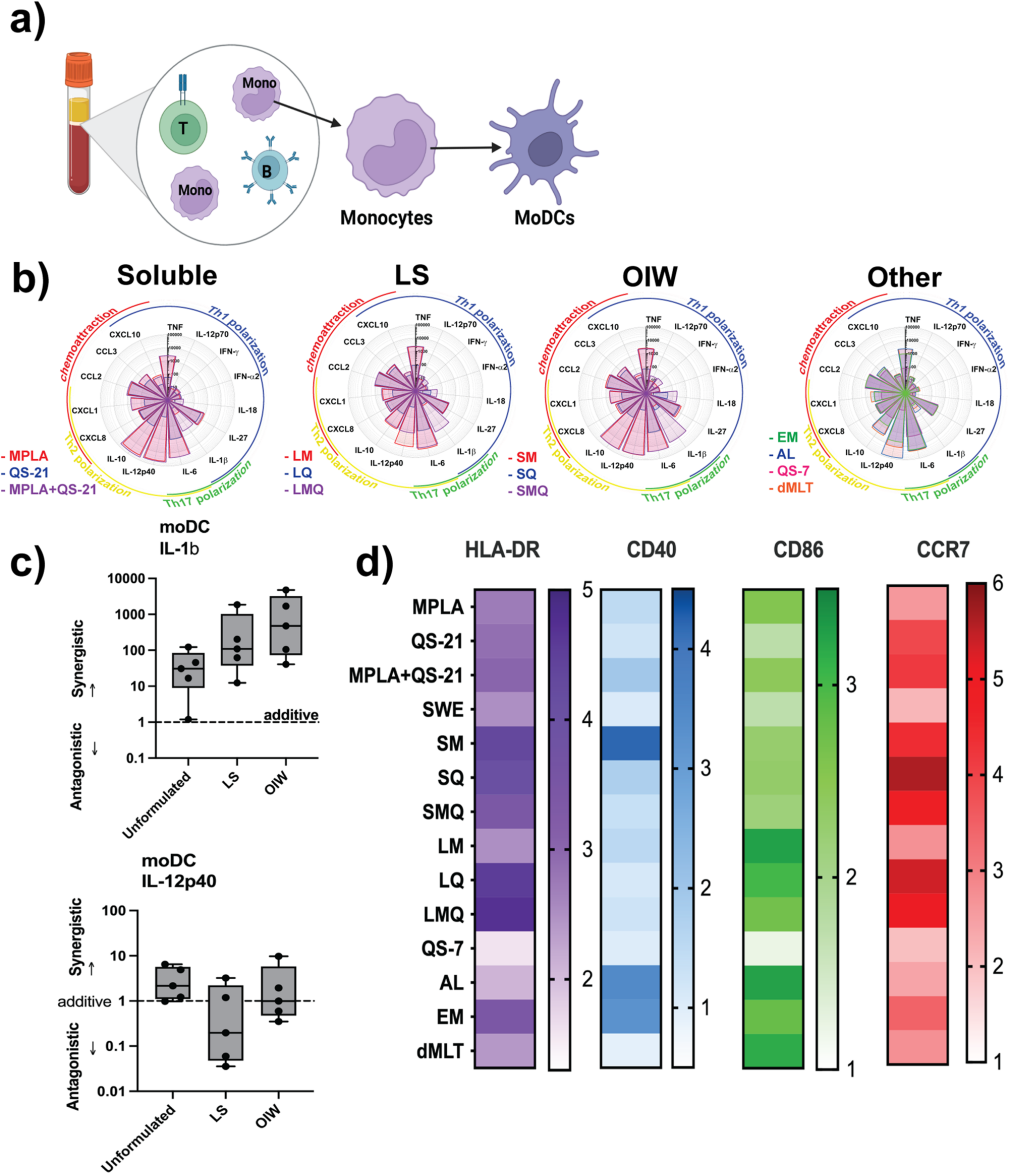
LS-based MPL, QS21 or combination formulations activate MoDCs to produce a Th1-polarizing cytokine response in monocyte-derived dendritic cell (MoDC) assay. MoDCs were stimulated with adjuvants at a 1:100 dilution for LNP, OIW, and Other, and 1:1000 dilution for Soluble formulations, before flow cytometric analysis of treatment-induced migrated cells and quantification of treatment-induced cytokine secretion. **a** Schematic representation of the monocyte-derived dendritic cell (MoDC) assasy. **b** Flower plots depict expression levels of 21 cytokines in pg/mL induced by treatment with adjuvants at their top concentrations. Dose-response curves show IL-1β production across a five-point dilution series (1/6 (v/v) each dilution, for all formulations) alongside a vehicle control. **c** Calculated D-values quantify the extent of synergy between MPL and QS-21 in each formulation type. D-values > 1 indicate antagonism, <1 indicate synergy, and =1 indicate additivity. (Box-whisker plots: centre line: median; bounds: 25th/75th percentile; whiskers; lowest/highest point). **d** Surface expression of four activation markers based on log10 MFI. Stars above a data point on flower plots and dose-response curves indicate statistical significance relative to vehicle control using a two-way repeated measures ANOVA and Dunnet’s method for multiple comparison testing. *N* = 5 for all assays. (*p < 0.05, **p < 0.01, ***p < 0.001).

Having found that MPL- and QS-21-containing adjuvants, and to a lesser degree 3M-052 and dmLT, drive differentiation of monocytes to a DC-like phenotype, we next assessed the effect of these adjuvants on human DCs. We stimulated MoDCs derived from the same donors with adjuvant formulations and cultured them in 10% autologous plasma (v/v) (Fig. 3a).

To describe the potential to promote differentiation of different T-helper subsets, we quantified the levels of secreted cytokines in response to adjuvant stimulation. While soluble MPL + QS-21 and OIW emulsions induced robust, predominantly Th2-polarizing cytokine responses, we found that MoDCs stimulated with MPL + QS-21 formulated in LS had a more Th1-biased response characterized by lower levels of IL-10 and IL-12p40 (Fig. 3b).

Most strikingly, formulations containing only MPL or QS-21 produced between one and three orders of magnitude less IL-1β than their co-formulated counterparts. To calculate the magnitude of this synergistic effect, we used a previously described adaptation of the Loewe method to measure the D-value for the dose-response curves. IL-1β was synergistically induced by the co-formulation of MPL and QS-21 across formulation types, with greater degrees of synergy in OIW emulsions and LS than in soluble formulations (Fig. 3c). Of note, IL-12p40 expression was antagonized by co-formulation in LS, while there was a purely additive effect of co-formulation in OIW emulsions and as soluble adjuvants—further highlighting the Th1-polarizing phenotype of LS formulation. Formulation of MPL and QS-21 in LS or OIW emulsions effectively activated DCs, inducing high levels of MHC-II and CCR7 expression on cell surface (Fig. 3d). Stimulation with MPL and QS-21 formulated in LS also induced robust expression of CD86, comparable to that induced by 3M-052-based formulations.

### Co-formulation of MPL and QS-21 in LS and OIW emulsions enables synergistic induction of a CD8^+^ T cell response in dendritic cell-T cell interface assay

**Fig. 4:**
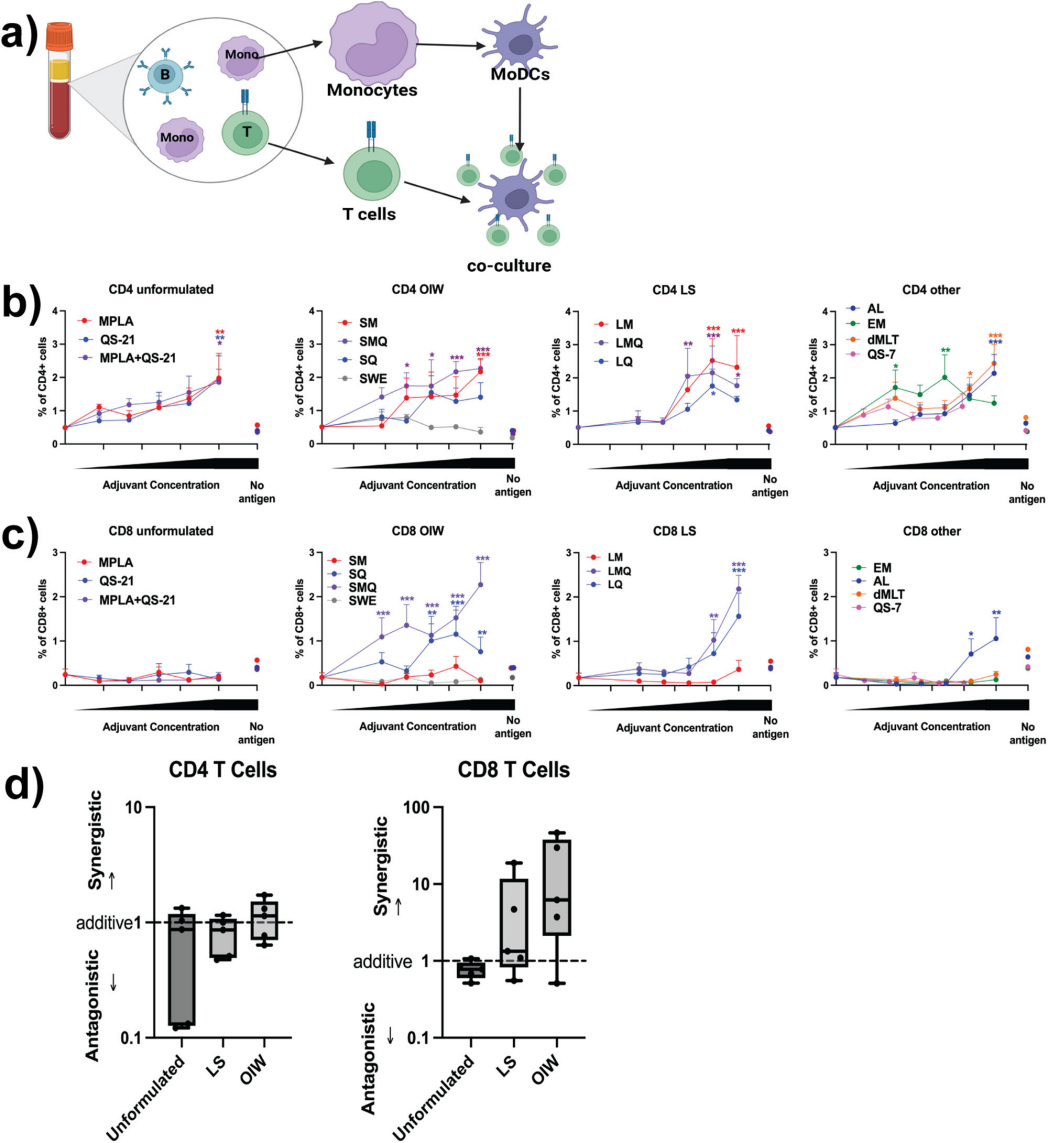
MoDCs stimulated with co-formulated MPL and QS-21 activate SARS-CoV-2 Spike protein-specific T cells in DTI assay. **a** Schematic representation of the DC:T cell interface (DTI) assay. **b**, **c** MoDCs were stimulated with 5 μg/mL SARS-CoV-2 Spike antigen, in the presence or absence of adjuvant formulations at a 1:100 dilution for LNP, OIW, and Other, and 1:1000 dilution for Soluble formulations, and subsequent 1/6 (v/v) dilutions for all formulations) before co-culture with autologous CD4^+^ or CD8^+^ T cells. Activated T cells were defined as CD4^+^, CD8^+^, CD25^+^, CD134^+^, or CD154^+^. Mean + SD are plotted for each concentration. **d** Calculated D-values quantify the extent of adjuvant interaction (i.e., antagonism, additivity, or synergy) between MPL and QS-21 in each formulation type. (Box-whisker plots: centre line: median; bounds: 25th/75th percentile; whiskers; lowest/highest point) Three data points were excluded (2 soluble, 1 OIW) because a curve could not be fit using the method described. Stars above a data point on dose-response curves indicate statistical significance relative to vehicle control, stars adjacent to vertical bars on dose-response curves indicate statistically significant differences between treatment groups at a given concentration, and stars above horizontal lines indicate statistically significant difference relative to a control with no antigen. Significance was calculated using a two-way repeated measures ANOVA with Tukey’s multiple comparison testing. *N* = 5 for all assays. (*p < 0.05, **p < 0.01, ***p < 0.001).

As antigen-presenting cells, DCs are a crucial link between innate and adaptive immunity. To evaluate the effect of the adjuvant formulations on antigen presentation by DCs, we modeled the process of antigen presentation using an in vitro DC:T cell interface assay (DTI, Fig. 4a). We stimulated donor-derived MoDCs with adjuvants for 24 h in the presence of SARS-CoV-2 Spike protein, and then cultured them with autologous CD4^+^ or CD8^+^ T cells for four days. Human DCs from nearly all formulations were able to activate autologous Spike-specific CD4^+^ T cells in a dose-dependent manner but with a different magnitude, with MPL-containing adjuvant formulations resulting in the highest percentage of activated antigen-specific CD4^+^ T cells (Fig. 4b). In contrast, only QS-21-containing OIW emulsions, LS, and AL induced robust activation of CD8^+^ T cells (Fig. 4c). Soluble QS-21-containing formulations induced no discernable CD8^+^ activation, nor did a 3M-052-based emulsion without Alum. Furthermore, we observed that relative to formulations with QS21 alone, co-formulation of QS-21 and MPL in OIW and LS induced a greater CD8^+^ response. The D-values for MPL and QS-21 co-formulation supported this observation, indicating a slight synergistic effect in LS and OIW emulsions (Fig. 4d).

### In vivo validation of adjuvanticity and tolerability

**Fig. 5:**
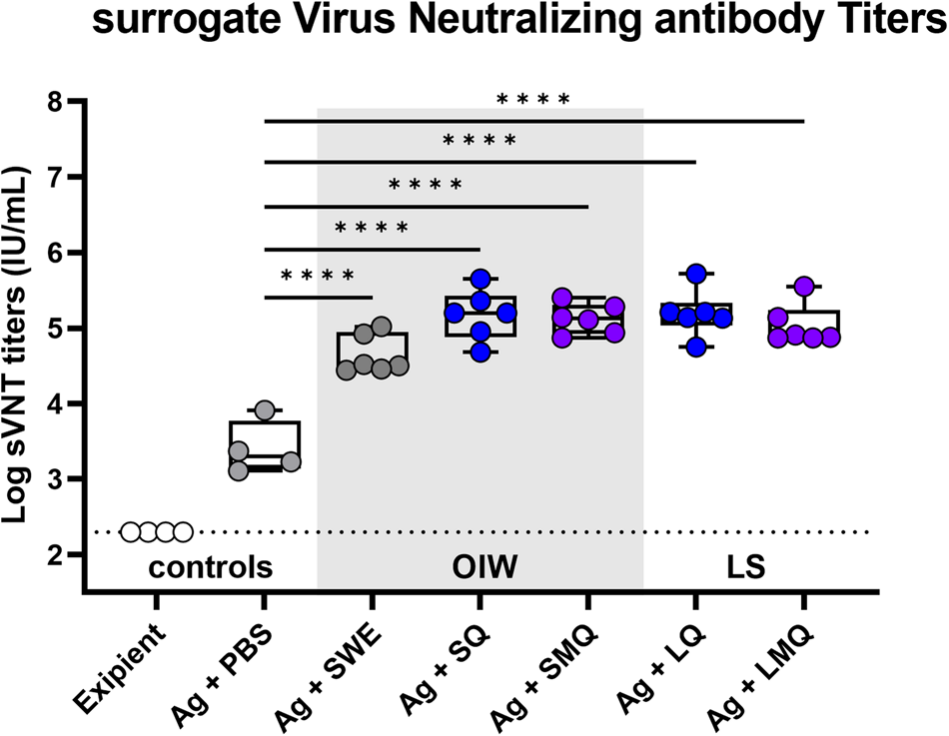
Immunization with SARS-CoV-2 Spike antigen-containing adjuvant formulations results in increased surrogate Virus Neutralization Titer (sVNT). Sera from C57BL/6J mice immunized on days 0 and day 21 with excipient, antigen (Ag) alone or formulations containing antigen and various adjuvants were collected on day 42. Surrogate virus neutralizing titers were quantified by measuring inhibition of RBD binding to human ACE2 receptor. (Box-whisker plots: centre line: median; bounds: 25th/75th percentile; whiskers; lowest/highest point) Significance was calculated using a two-way repeated measures ANOVA with Tukey’s multiple comparison testing. *N* = 4–6 (*p < 0.05, **p < 0.01, ***p < 0.001), ****p < 0.0001).

Immunization of mice with admixtures of recombinant SARS-CoV-2 Spike protein antigen (wild type) with, SWE, LQ, LMQ, SQ, or SMQ adjuvants demonstrated that antigen formulations containing an adjuvant induced significantly higher sVNT antibody titers than those observed in excipient and non-adjuvanted antigen groups (Fig. 5). These data indicate that these antigen formulations containing liposomal or emulsion-based adjuvants induced robust humoral antibody responses in vivo. In an independent control experiment, similar observations were made through quantification of anti-Spike antibodies by ELISA. Co-formulation of Spike protein with LQ, LMQ, SQ, or SMQ adjuvants resulted in significantly amplified spike-specific IgG, IgG1, and IgG2a over vehicle and non-adjuvanted controls on days 28 and 42 post-prime vaccination (Supplementary Fig. 4A, B). Relative ratios of IgG2a over IgG1 can infer immune polarization states for Th1 and Th2 responses, respectively^21^. Spike antigen alone induced a Th2-associated response, while a relative shift towards Th1 polarization was observed in LS formulations, and trends of greater Th1 polarization were observed with OIW containing formulations (Supplementary Fig. 4C, D). Formulations were well-tolerated, and weight change was not significantly impacted, as no mouse lost more than 5% of starting weight (Supplementary Fig. 4E).

## Discussion

Our findings that the liposomal co-formulation of MPL and QS-21 acted synergistically in multiple readouts and induced the development of antigen-specific T-helper 1 cells and CD8^+^ T cells provide an understanding of the underlying MOA of adjuvant formulations that boost immune response through vaccination. We used distinct but complementary human in vitro platforms to model innate and adaptive immune responses and made observations that could predict in vivo activity and advance the development of safe and effective low-cost vaccines. The formulation type also determines overall reactogenicity and polarity of the innate and adaptive immune responses, and we found LS formulation of adjuvants to be less reactogenic in the whole blood assay platform. QS-21 was the primary adjuvant driving receptor upregulation in the MoDC assay, and the production of Th1-polarizing cytokines was mainly associated with the LS formulation. We also found synergistic induction of IL-1β with LS and OIW formulations, and antagonism with Th2-polarizing cytokine IL-12p40 with the LS co-formulation of MPL and QS-21. QS-21-containing formulations promoted antigen processing and cross-presentation on MHC in the DC-T cell interface assay, and QS-21 was the adjuvant responsible for driving the CD8 response.

The observed human-specific in vitro bioactivity of formulations was validated in vivo, in a murine model. Specifically, significant adjuvanticity compared to antigen-alone was observed across both liposomal and oil in water formulations (Fig. 5), indicating that the immunostimulatory capacity observed in humans associated with increased adjuvanticity in vivo. LS and OIW formulations induced more Th1 polarization-associated relative IgG2a:IgG1 ratios (Supplementary Fig. 4C, D), further supporting in vitro human observations. Importantly, tolerability of vaccine formulations was established, with low TNF, and non-detectable systemic IL-6 or IL-1β induction, each of which have been associated with aberrant vaccine reactogenicity^22–25^, alongside no mice exceeding 5% weight loss of starting weight (Supplementary Fig. 4E). Formulations were therefore beneficial in amplifying immunogenicity in vivo and were well tolerated.

The ability of AS-01 to increase antigen presentation to antigen-specific immune cells enhances vaccine immunogenicity and efficacy, providing durable protection against targeted pathogens^19^. The mechanistic and synergistic action of MPL + QS-21 liposomal co-formulation that we describe is comparable to the MOA of AS-01 previously described^26–28^. The similarity of MPL + QS-21 liposomal co-formulation to AS-01 may advance opportunities to develop low-cost vaccines and vaccines for vulnerable populations such as children, older adults, and immunocompromised individuals using adjuvant formulations produced for global open access. This approach could make a significant public health impact globally, especially when we are faced with epidemics and pandemics, by controlling the spread of infections via rapid and equitable distribution of vaccines. The broad cytokine expression profile shown by the adjuvants could potentially be useful in vaccines targeted against pathogens displaying antigenic drift, strain variations or both, such as influenza viruses.

While featuring multiple strengths and complimentary approaches, our study also has several limitations. One limitation of our study is the small sample size, and therefore we could not stratify by demographic differences affecting immune responses. We focused our study on the MOA of individual and combination adjuvants, with each of the participants serving as both the test and control condition in the in vitro platforms, leveraging paired analyses and capturing participant variability across treatment groups. In addition, we did not pursue to establish the kinetics of cytokine secretion, such as IL-10 and IL-12p40, though we used five different concentrations of the adjuvant formulations at single time points to assess the immunoregulatory role of cytokines. It is unclear if these cytokines are consistently released over time or if the secretion reduces after the initial production. With the selected concentrations, we were unable to demonstrate a strong response of dmLT that has shown to induce IL-1β and other cytokines in previous studies^29–32^. We chose concentration ranges that would minimize toxicity and allow clean in vitro study. However, toxicity and pyroptosis could potentially contribute to in vivo efficacy. In vivo evaluation identified tolerability and biological activity of selected formulations, adjuvanting greater humoral anti-spike immunity. However, the in vivo adjuvanticity and tolerability profiles require further investigation in other species, including humans,

In sum, we have leveraged a range of innovative human in vitro assays, paired with in vivo murine validation, to define novel combinatorial effects of adjuvant formulations. The mechanistic understanding of adjuvant activity on immunity that our study provides would inspire further investigations on immune ontogeny and lead to the development of vaccines for global access.

## Methods

### Adjuvants and formulations

The following adjuvants were used at the dilutions noted in the figure legends: SWE (squalene oil-in-water (OIW) emulsion, stock 4.1% squalene, Seppic, France), MPL (3D-6A PHAD, stock 1 mg/mL in DMSO, Avanti Polar Lipids, Alabaster, AL), QS-21 and QS-7 (stock 1 mg/mL in DMSO, Desert King, San Diego, CA), 3M-052 absorbed to 2 mg/mL Alum (AL030, stock 0.12 mg/mL, 3 M Corporate Research and Materials Laboratory (St. Paul, MN) and Access to Advanced Health Institute (AAHI, Seattle, Washington)), 3M-052 in 4% OIW (EM128, stock 0.12 mg/mL, 3 M/AAHI), and dmLT (stock 1 mg/mL in 700 μg of protein in 42.7 mM sodium phosphate, 10.7 mM potassium phosphate, 82 mM NaCl, 5% lactose, supplied by PATH (Seattle, Washington), manufactured by Walter Reed Army Institute of Research Pilot Bioproduction Facility (Silver Spring, MD)). LM, LQ, LMQ (liposomal (LS) formulations of MPL, QS-21 and MPL + QS-21 respectively), SM, SQ, and SMQ (OIW emulsions of MPL, QS-21 and MPL + QS-21 respectively) were obtained from the Vaccine Formulation Institute (VFI, Plan-les-Ouates, Switzerland). Non-MPL-containing stocks were confirmed to be free of endotoxin at the concentrations used (<1 EU/mL) using a limulus amoebocyte lysate (LAL) assay in accordance with the manufacturer’s protocol (Charles River, Wilmington, MA). Unformulated MPL, QS-21, and QS-7 were prepared in DMSO (Fisher Scientific, Hampton, NH), and DMSO controls were used as vehicle controls. Dynamic light scattering was performed on all formulations using a Zetasizer Nano ZS (Malvern Panalytical, Malvern, United Kingdom) to measure size, polydispersity index, and surface zeta potential over time to ensure formulation stability (Supplementary Fig. 1).

### Blood collection

Peripheral blood samples from healthy study participants (*n* = 5; 26–45 years old) was collected after written informed consent was obtained in accordance with the Declaration of Helsinki and as approved by the Ethics Committee of Boston Children’s Hospital (BCH) (protocol number X07-05-0223). All participants were fully immunized against SARS-CoV-2 (>2 weeks prior) with one of the three FDA Emergency Use Authorized vaccines (Pfizer/BioNTech, Moderna, Janssen) with approval from the Ethics Committee of Boston Children’s Hospital, Boston, MA (protocol number X07-05-0223). Samples were de-identified, anti-coagulated with 20 U/mL pyrogen-free heparin sodium (Fresenius Kabi, Bad Homburg, Germany), and processed within 2–4 h.

### Whole blood assay (WBA)

We adapted a previously described method to measure adjuvant activity in whole blood^33^ Briefly, whole blood was mixed 1:1 with sterile RPMI 1640 medium (Gibco, Ward Hill, MA, USA) and 180 µL of this was added to each well of a 96-well U-bottom plate containing 20 µL of freshly prepared adjuvant formulations at 10x the final concentration in RPMI 1640. The 200 µL/well suspensions were gently mixed by pipetting and were incubated at 37 °C for 24 h in a humidified incubator with 5% CO_2_. After incubation, the plate was centrifuged for 3 min at 500 × *g*. Supernatants were collected through pipetting without disturbing the cell pellet and were frozen at −80 °C until cytokine multiplex was run. Partial lysis of erythrocytes was obtained by incubating the cell pellet with BD FACS lysing solution (BD Biosciences, San Jose, CA, USA). Cells were washed three times with DPBS (Gibco, Ward Hill, MA, USA), fixated in 1% paraformaldehyde (Thermo Fisher Scientific, Ward Hill, MA, USA), and stored at 4 °C until flow cytometry analysis.

### Isolation of mononuclear cells, monocytes and T cells, and generation of MoDCs

Mononuclear cells and monocytes were isolated as follows^34^. Heparinized blood from adult donors was centrifuged at 500 × *g* for 10 min. The upper layer of platelet-rich plasma was removed and further centrifuged at 3000 × *g* for 10 min. The platelet-poor plasma was stored at 4 °C for use in culture. Blood was restored to its original volume by adding DPBS and was carefully layered onto a Ficoll-Paque gradient (Cytiva, Marlborough, MA, USA). The mononuclear cell fraction was collected after centrifugation for 30 min at 1000 × *g* and was washed twice with DPBS. Monocytes were isolated from this cell fraction through positive selection with magnetic CD14 MicroBeads (Miltenyi Biotec, Auburn, CA, USA) in accordance with the manufacturer’s instructions. CD8^+^ and CD4^+^ T cells were then sequentially isolated from the remaining cell fraction in the same way, using CD8^+^ and CD4^+^ MicroBeads in accordance with the manufacturer’s instructions. CD14^+^ monocytes were cultured in a humidified incubator at 5% CO_2_ at 37 °C in 75 cm^2^ tissue culture flasks at a concentration of 10^6^ cells/mL of RPMI 1640 supplemented with 1% penicillin-streptomycin (Gibco, Ward Hill, MA, USA), 10% FBS, 0.1% IL-4, and 0.1% GM-CSF (Miltenyi Biotec, Bergisch Gladbach, Germany) for five days. CD8^+^ and CD4^+^ T cells were frozen in 10% FBS (Gibco, Ward Hill, MA, USA), 10% DMSO and 80% RPMI, and were stored at −80 °C until use. After five days of culture, immature monocyte-derived dendritic cells (MoDCs) were harvested by removing the loosely adherent cell fraction through gentle pipetting.

### MoDC assay

MoDCs were suspended in fresh MoDC culture medium at a concentration of 1.11 × 10^6^ cells/mL, and a volume of 180 µL (200,000 cells) was plated into each well of a 96-well U-bottom plate containing 20 µL of freshly prepared adjuvant formulations at 10× the desired final concentration. Suspensions were mixed by gentle pipetting and the cells were then cultured for 24 h in a humidified incubator at 37 °C with 5% CO_2_. Cells were then centrifuged for 3 min at 500 × *g*, and supernatants were harvested and stored at −80 °C for use in further functional assays. Cells were washed with DPBS and fixated for flow cytometry.

### MoDC:T cell interface assay (DTI)

DTI was performed as follows^16^. MoDCs generated as described above were suspended in RPMI 1640 supplemented with 1% penicillin-streptomycin (Gibco, Ward Hill, MA, USA), 10% FBS, 0.1% IL-4, and 0.1% GM-CSF. 25,000 cells were added per well in 90 µL to a 96-well U-bottom plate containing 10 µL of adjuvant formulations at 10× the desired final concentration in the presence or absence of recombinant SARS-CoV-2 Spike protein (Thermo Fisher Scientific, Ward Hill, MA, USA). Suspensions were mixed by gentle pipetting and cultured for 24 h. On the same day, CD4^+^ and CD8^+^ T cells were thawed and resuspended in RPMI 1640 with 10% FBS and 1% penicillin-streptomycin at a concentration of 3.33 × 10^6^ cells/mL. T cells were cultured for 24 h in a six-well plate with 3 mL per well, and then harvested by gentle pipetting, resuspended at a concentration of 2.5 × 10^6^ cells/mL. 100 µL of either CD4^+^ or CD8^+^ T cells was added to each well of the respective MoDC plate, and the co-culture was incubated for four days. Cells were then centrifuged for 3 min at 500 × *g*. Supernatants were harvested and stored at −80 °C for use in further functional assays. Cells were washed with DPBS, fixated in 1% PFA, and stored at 4 °C for flow cytometry.

### Human tissue construct (HTC)

A human tissue construct model of monocyte extravasation and in-tissue autonomous differentiation was generated as we have previously described^13^. Briefly, Endotoxin-free human type I collagen Advanced Biomatrix^TM^ (San Diego, CA) cushions were cast in 96-well microtiter plates (Costar round bottom, Thermo Fisher Scientific Inc., Ward Hill, MA, USA). Human type I collagen cushion solution was prepared by mixing 10x M199 media (Gibco, Ward Hill, MA, USA), 0.1 N NaOH, and human collagen (3 mg/mL) at a proportion of 1:5:8 respectively. 70 µL of this solution was applied to each of the inner 60 wells of a 96-well flat-bottom plate using a repeat pipette, and 200 µL of HBSS (Gibco, Ward Hill, MA, USA) was added to the 36 outer wells to act as an evaporation barrier. The plate was incubated at 5% CO_2_/37 °C for 24 h to congeal. Meanwhile, using Trypsin-EDTA and the same M199 media containing 50% FBS and 1% Penicilin/Streptomycin/Glutamine (Gibco, Ward Hill, MA, USA), 85–90% confluent human umbilical vein endothelial cells (HUVEC) cultures (as assessed with an inverted microscope) were passed to larger (150 cm^2^) vented cap tissue culture flasks pre-coated with human fibronectin (0.5 mg/mL, Biomedical Technologies Inc. Stoughton, MA) and incubated at 5% CO_2_/37 °C. Ten minutes before seeding endothelial cells, collagen cushions were prepared by adding 100 µL of M199 with 1% Penicilin/Streptomycin/Glutamine (Gibco, Ward Hill, MA, USA) for 10 minutes before aspirating out and adding 20 µL of 0.5 mg/mL human fibronectin. Typically, one T150 flask of confluent HUVECs contained sufficient cells to coat two cushion plates (120 wells). Prior to seeding, excess fibronectin was aspirated out and cells from one confluent T150 flask were resuspended in approximately 13 mL of media to be dispensed as 100 µL per well (120 wells). Plates were incubated at 5% CO_2_/37 °C until confluency of monolayer. Monolayer integrity, confluence, and morphology of endothelial cells were assessed by inverted microscopy using phase contrast at 4× magnification (Nikon TS100 inverted microscope EVOS XL Core Imaging System (Fisher Scientific, Hampton, NH, USA)). Only 100%-confluent TCs were used for testing. CD33^+^ cells from each donor were plated on top of autologous platelet-poor plasma. The adjuvant formulations were immediately added to the appropriate wells from a pre-prepared dilution plate. The plates were incubated for 48 h at 37 °C in 5% CO_2_. After incubation, representative images were taken, and supernatants were collected without disturbing the endothelial layer. The reverse-transmigrated cells were then resuspended in DPBS and transferred to a fresh 96-well U-bottom plate. 10 µL of sample was removed for counting in a hemocytometer after dilution in Trypan Blue (Gibco, Ward Hill, MA, USA). Remaining cells were washed again in DPBS, fixated in 1% PFA (Fisher Scientific, Hampton, NH, USA), and stored at 4 °C for flow cytometry.

### Cytokine multiplex

Cytokine profiles of supernatants derived from whole blood assay, MoDC assay, DTI assay, and HTC assay were analyzed using a multianalyte fluorescent bead-based array (Luminex Corp., Austin, TX, USA). Quantification of cytokines was done using a custom built 21-plex kit using the Milliplex HCYTA-60K Human Cytokine/Chemokine/Growth Factor Panel A (Millipore, Merk, Darmstadt, Germany). This customized kit included CXCL8, CXCL10, GRO, IFNa-2, IFNγ, IL-1β, IL-2, IL-3, IL-4, IL-5, IL-6, IL-10, IL-12p40, IL-12p70, IL-13, IL-17, IL-18, IL-27, MCP-1, MIP-1a, and TNF, and was run according to the manufacturer’s instructions. Sample fluorescence data was collected using a Flexmap 3D analyzer running xPONENT software version 4.2, and results were fit to a 5-point log curve and converted into pg/mL values using manufacturer-provided standard solutions and Milliplex Analyst software version 5.1.

### Flow cytometry

Flow cytometry was used to identify and characterize cell subpopulations following whole blood assay, MoDC assay, DTI assay, and HTC assay. Flow cytometry was performed using an LSRFortessa flow cytometer (Becton Dickinson) and analyzed with FlowJo software version 10. Cells were stained for 30 min at 4 °C in the dark with panels which used the following antibodies: anti-CD14-PE (Clone M5E2, catalog (cat)# 557154), anti-CD56-PerCP-Cy5.5 (Clone B159, cat# 560842), anti-CD20-PE-Cy7 (Clone L27, cat# 335793), anti-CD86-AF700 (Clone 2331, cat# 561124), anti-CD197-PE-Cy7 (Clone 3D12, cat# 557648), anti-CD4-V450 (Clone RPA-T4, cat# 560345), anti-CD8-V450 (Clone RPA-T8, cat# 561426), anti-CD134-PE (Clone L106, cat# 340420), anti-CD154-APC (Clone 89-76, cat# 648887), anti-CD25-FITC (Clone M-A251, cat# 555431), anti-CD16-FITC (Clone B73.1, cat# 561308), anti-CD40-FITC (Clone 5C3, cat# 555588) (BD Biosciences, East Rutherford, NJ), anti-CD3e-AF647 (Clone UCHT1, cat# A51001) (Invitrogen, Waltham, MA), anti-CD123-APC-eFluor780 (Clone 6H6, cat# 47-1239-42) (eBioscience, San Diego, CA), anti-CD1c-PB (Clone L161, cat# 331508), and anti-HLA-DR-BV605 (Clone L243, cat# 307639) (Biolegend, San Diego, CA). All antibodies were added at 1:25 (v/v) dilution. Cells were then washed and resuspended in DPBS prior to data acquisition. All representative gating strategies are shown in Supplementary Fig. 3.

T-SNE analysis in Fig. 2 was run using Flowjo, All flow cytometry samples from all treatments combined were used to map t-SNE populations. Uncompensated parameters were selected, additional parameters were set to 1000 iterations, complexity 30, and a learning rate of 7% total events mapped. Vantage point tree k-nearest neighbors (KNN) algorithm was used in combination with Barnes-Hut gradient algorithm.

### Mouse immunization

All animal studies complied with all relevant ethical regulations for animal testing and research set by the Swiss Federal Law on the Protection of the Animals and the Association for the Assessment and Accreditation of Laboratory Animal Care International (AAALAC) and have received ethical approval from the animal care and use committee (IACUC) regulatory committee at Boston Children’s Hospital (protocol number 00001573). Animals were co-housed with food (isopro RMH3000 irradiated) and water ad libitum. After completion of all experiments, animals were euthanized following institutional guidelines, specifically by regulated exposure to CO2, with confirmation by paw-pinch and cervical dislocation. For evaluation of surrogate virus neutralization titer (sVNT), six- to eight-week-old female C57BL/6J mice (Charles River) were immunized intramuscularly (hind leg) on days 0 and 21 with 50 µL of adjuvanted SARS-CoV-2 Spike antigen formulations, or controls including excipient and SARS-CoV-2 Spike antigen alone. Blood samples were collected on days 20 and 42, incubated at RT for 2 h to allow clot formation and centrifuged at 1000 × *g* for 10 min. Serum layers were then collected and stored at −80 °C prior to analysis. SARS-CoV-2 spike receptor binding domain antibodies capable of blocking human ACE2 receptor binding were detected in sera using sVNT, (Genscript, Cat# L00847) specific for wild-type RBD (Z03594) according to the manufacturer’s instructions (Genscript,). Briefly, sera and control samples were prediluted before being mixed 1:1 with an HRP-RBD solution. After 30 minutes incubation at 37 °C, 100 µL of this preparation was added to the capture plate pre-coated with ACE2 human cell receptor and incubated for 15 min at 37 °C. After four washes, a TMB solution was added and the plate was incubated for 15 min at RT. The reaction was quenched with 1 M sulfuric acid and absorption was read at 450 nm using a microplate reader.

A control cohort of animals was immunized with institutional animal care and use committee (IACUC) regulatory approval at Boston Children’s Hospital. Adjuvant formulations as indicated in Supplementary Fig. 4 were co-formulated with SARS-CoV-2 Spike antigen, for analysis of anti-SARS-CoV-2 Spike antibodies by ELISA following immunization. Six- to eight-week-old BALB/cJ mice (Jackson Laboratories), were intramuscularly immunized (IM) in the caudal thigh with adjuvanted formulations as indicated, containing 1 µg of recombinant wild-type Spike protein (Wuhan human-1 isolate, GenBank MN90894, M1-Q1208), produced in HEK293 cells^35^. Mice were immunized following a prime (day 0), boost (day 14) immunization schedule. At 24 h post-prime and post-boost immunization, blood was collected in heparinized capillary tubes by retro-orbital (RO) bleed, stored on ice, and plasma isolated by centrifugation (500 × *g* for 10 min, room temperature) to evaluate the degree of reactogenicity-associated analyte induction. IL-6, TNF, and IL-1β were quantified by ELISA following the manufacturer’s recommendations (Invitrogen, 88-7064 (IL-6), 88–7324 (TNF), 88–7013 (IL-1β)). At 28 and 42 days post-prime immunization non-heparinized capillary tubes were used to collect blood by RO bleed and subsequently collect serum was collected by centrifugation within 2 h (1500 × *g*, 7.5 min). Serum was then transferred to a new microcentrifuge tube, and centrifuged again to maximize separation from clot, and increase serum volume collection. Anti-spike antibody quantification was performed as described previously^35^. Briefly, high-binding flat 96-well plates were coated overnight with 25 ng Spike antigen per well. After washing with 0.05% Tween20 in PBS plates were blocked with 1% BSA in PBS for 1 h, with incubation at room temperature (RT). Following an initial dilution of 1:100, 10 serial fourfold dilutions were applied to spike-coated plates for 2 h (RT). Following three washes, a 1 h incubation with horse radish peroxidase anti-mouse IgG, IgG2a, and IgG1, and another five washes, wells were incubated 5 min with tetramethylbenzidine (TMB; BD biosciences OptEIA Substrate Solution), then were inactivated with 2 N H_2_SO_4_. 450 nm absorbances were compared to blank, and interpolated titers were calculated based on the threshold crossing three times per-plate median blank values, and non-responsive samples were assigned a value of half lower limit of detection, 50, for statistical comparison.

### Statistical analyses

Statistical analyses were performed using GraphPad Prism version 9.3.1 and R (version 4.2.1) with RStudio (version 2023.03.1 + 446). Dose-response curves were compared using 2-way repeated measures ANOVAs, and multiple comparison testing was performed using either Dunnet’s Method when comparing against the vehicle only or Tukey’s method when making comparisons across additional groups. A normal two-way ANOVA with Tukey’s correction for multiple testing was used to analyze the HTC results as well as sVNT and antibody quantification results, as comparisons were only made at a single concentration. Synergy was calculated using an adaptation of the Loewe method of additivity^34^. D values greater than 1 were considered antagonistic, D values equal to 1 were considered additive, and D values less than 1 were considered synergistic. Humoral immunity from mice was evaluated for normality by Shapiro-Wilk followed by Kruskal-Wallis and two-sided Wilcoxon tests. Flower plots were generated with Grapher v15.2.311. Graphical illustrations (Figure panels 1A, 2A, 3A, 4A) were generated with Biorender.com under publication agreement JV25VQMXXO.

### Reporting summary

Further information on research design is available in the Nature Research Reporting Summary linked to this article.

### Supplementary information

REPORTING SUMMARY

Supplementary Figures 1-4

## Data Availability

Cytokine multiplexing data were archived on ImmPort (https://immport.niaid.nih.gov/home) under accession number **SDY2394**. All other datasets generated are available from the corresponding author upon reasonable request.
