# Supplementary material for: Human in vitro modeling of adjuvant formulations demonstrates enhancement of immune responses to SARS-CoV-2 antigen
Source: NPJ Vaccines. 2023 Oct 26;8:163. doi: 10.1038/s41541-023-00759-y (PMC10603059; doi:10.1038/s41541-023-00759-y)
Supplement: Supplementary file 2 — Supplementary Figures 1-4 [file 41541_2023_759_MOESM2_ESM.pdf]

Supplementary Figure 1

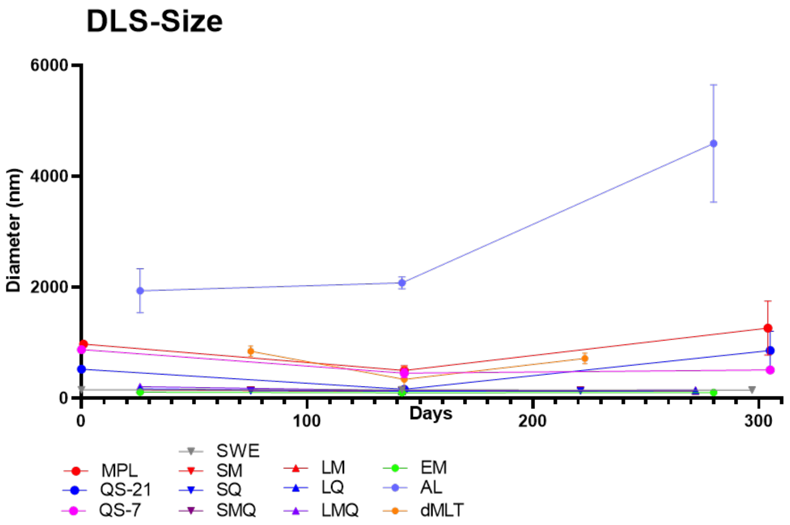

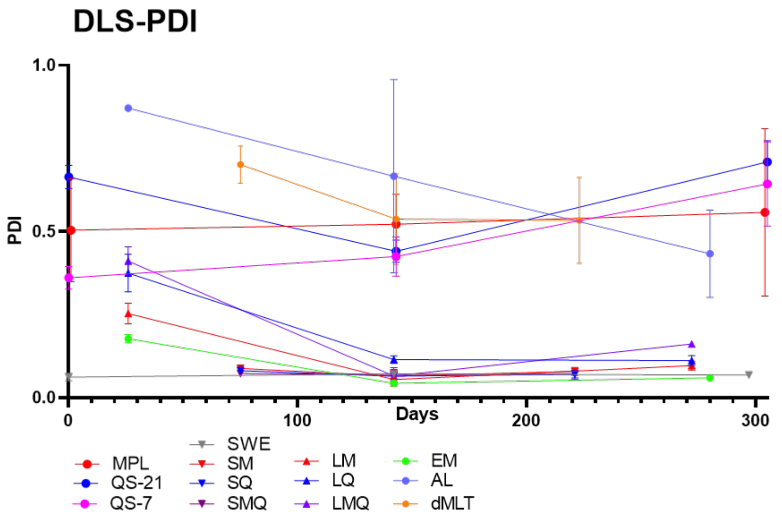

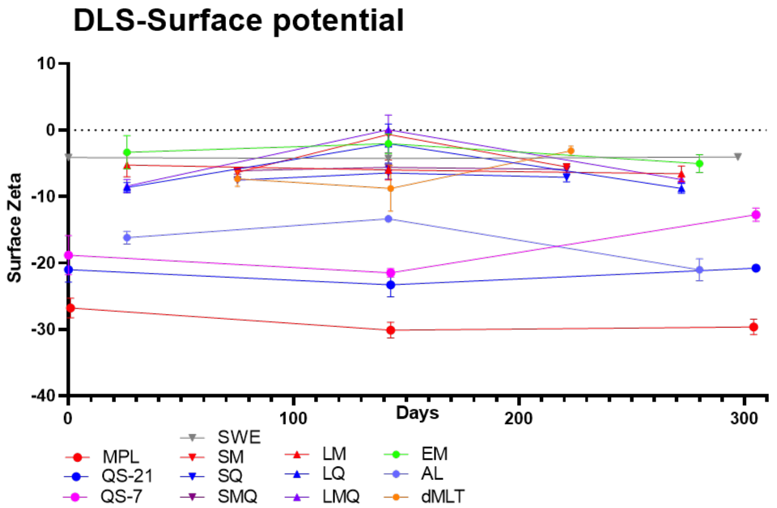

Size and surface potential determination of adjuvant formulations. All formulations were routinely tested for changes in size, polydispersity index, or surface potential by Dynamic light scattering (DLS). Changes were quantified over a period of 300 days of storage at 4C, with the exception of unformulated MPLA and QS-21 (-20C). All points are mean+SD.

Supplementary Figure 2

a) WBA

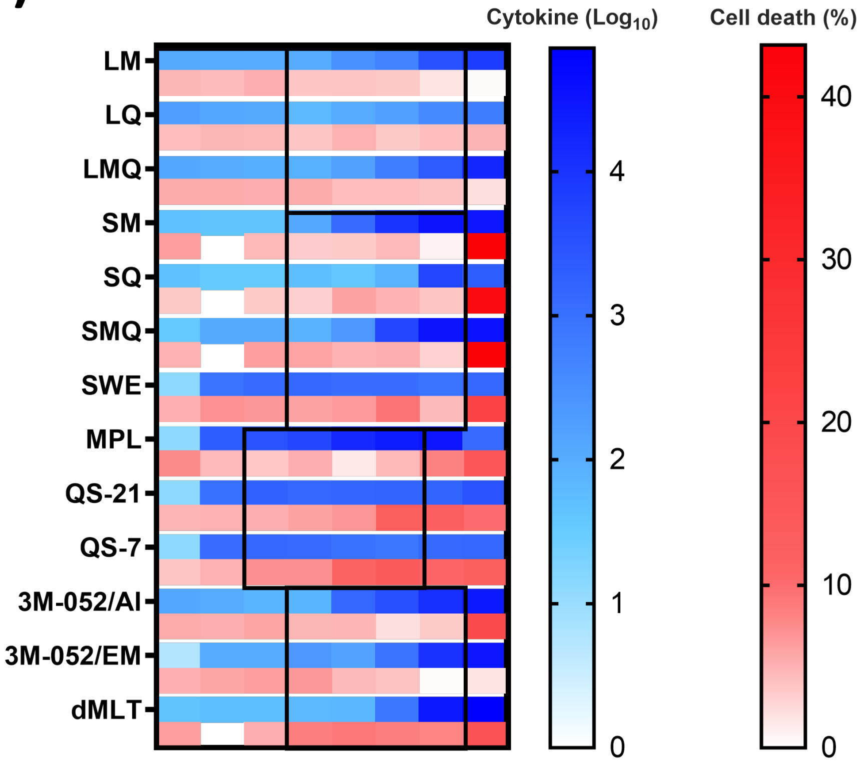

b) MoDC

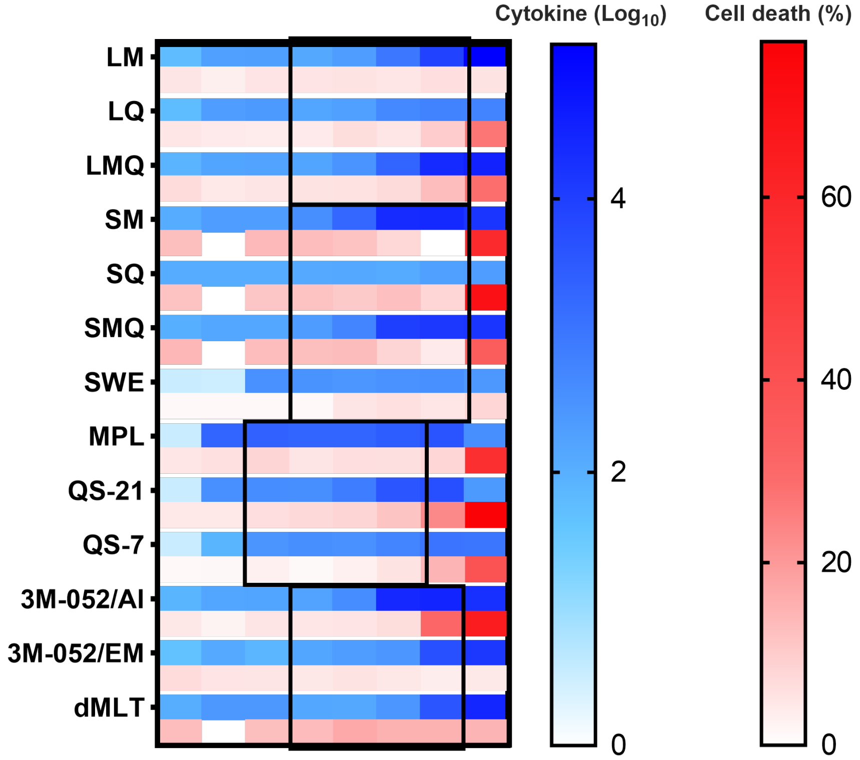

Activity titration of adjuvant formulations. All formulations were tested for optimal activity with minimal cytotoxicity as described in the Methods section. Cytotoxicity was represented by red heat maps, and activity is represented by blue heat maps. Black boxes represent concentration ranges for each adjuvant formulation that were used for further evaluation throughout the manuscript.

Supplementary Figure 3

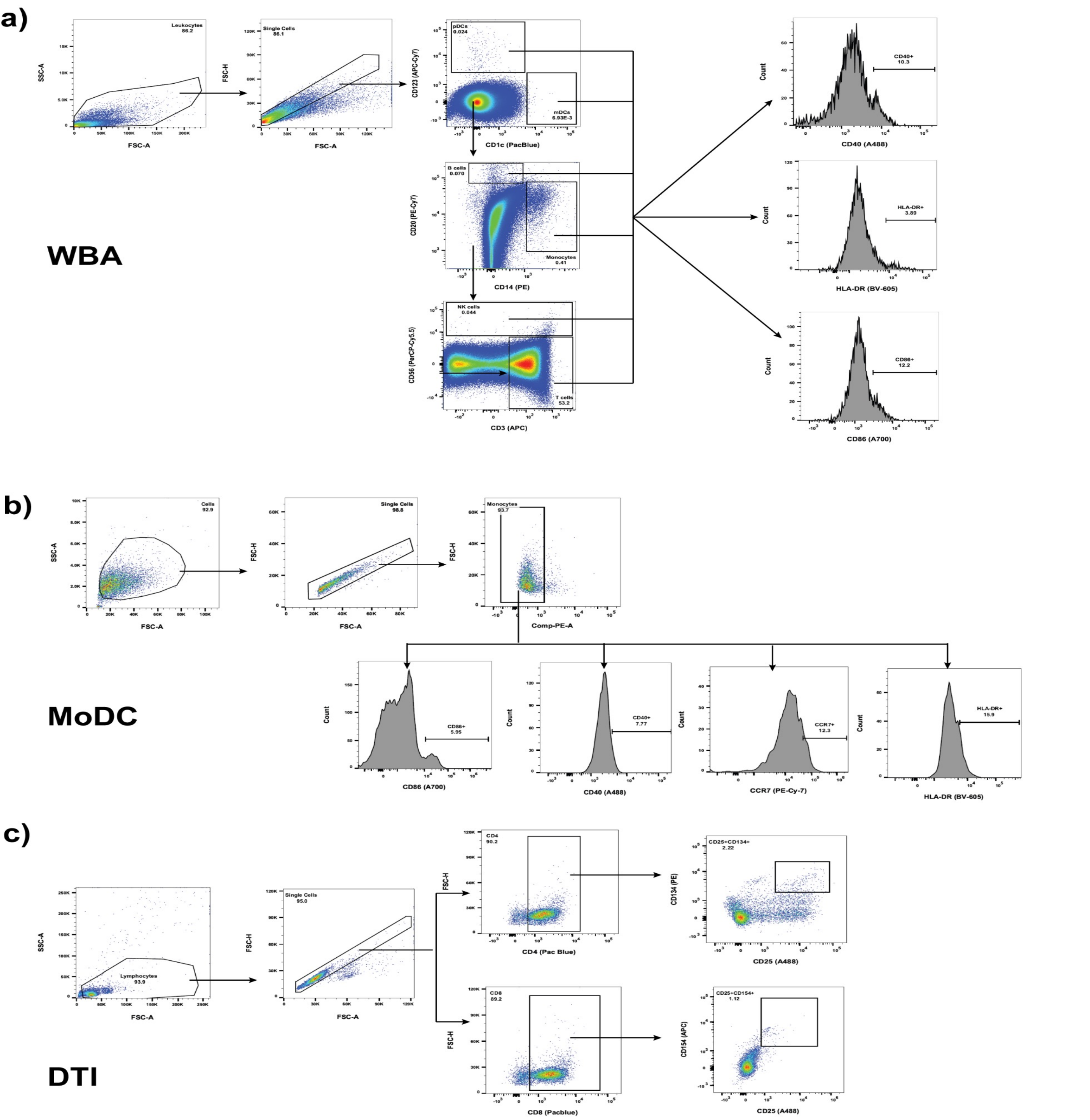

Representative Gating strategies. After flow cytometric acquisition, populations of interest were gated as illustrated in the representative images shown. WBA: Monocytes, NK cells, T cells, B cells, mDC, and pDCs were considered positive for CD14, CD56, CD3, CD20, CD11c, and CD123, respectively. Each population was subsequently assessed for positivity of activation markers CD40, HLA-DR, and CD86. MoDC cultures were selected for absence of CD14, and subsequently assessed for positivity of activation markers CD40, HLA-DR, CCR7, and CD86. DTI cultures contained MoDCs co-cultured 1:10 with either purified CD4 or CD8 T cells. T cells were identified and selected through expression of CD4 or CD8. T cells were considered to be activated in an antigen-specific manner when they co-cexpressed CD25 and CD134 (CD4 T cells), or CD25 and CD154 (CD8 T cells).

Supplementary Figure 4

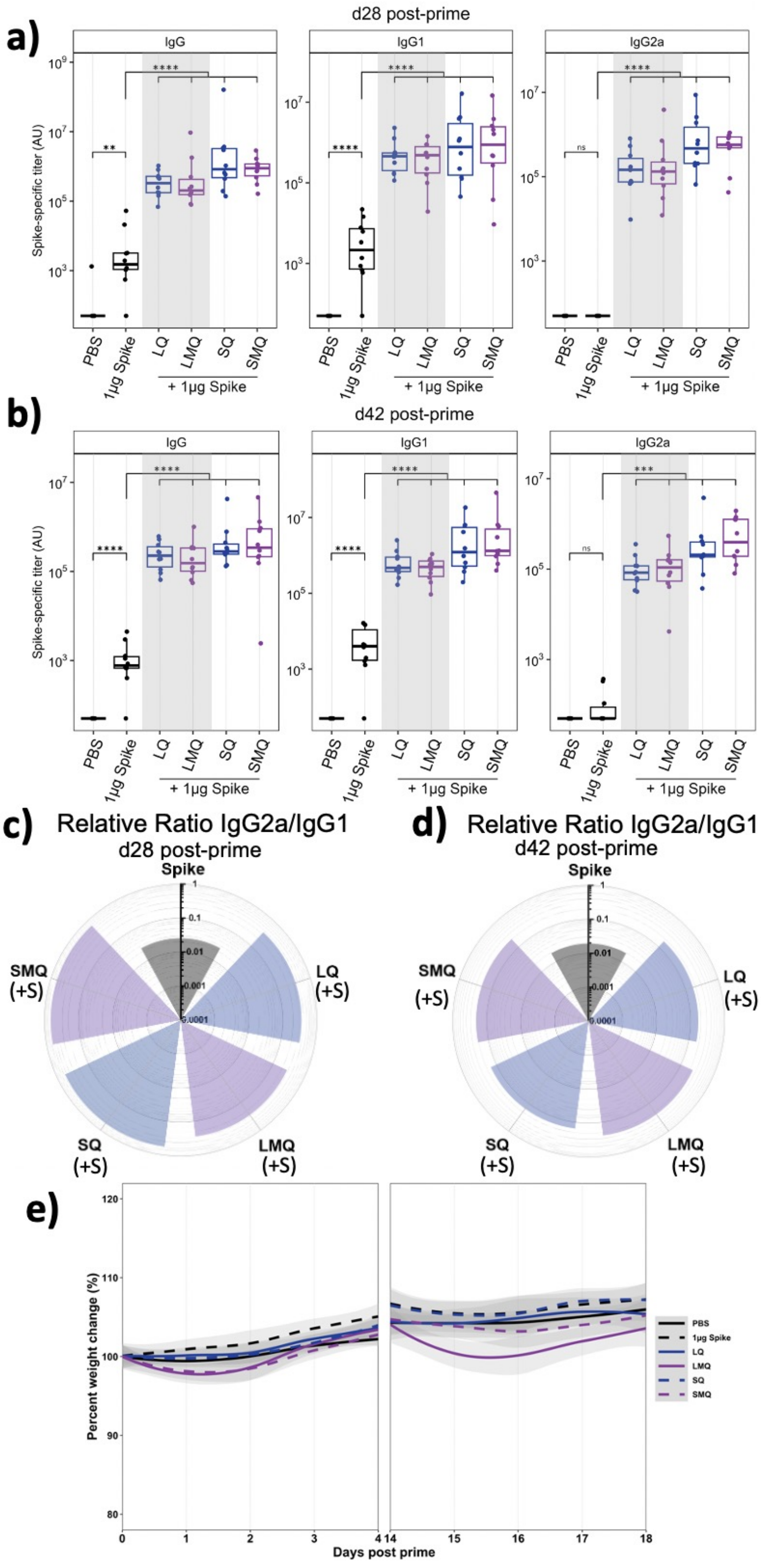

MPLA and QS-21 liposomal and oil-in-water formulations adjuvant murine humoral immunity to Spike antigen. Adult mice (6-10 weeks) were intramuscularly immunized following a 14 day separate prime-boost schedule. Injectant included 50 µL of vehicle control ('PBS'), 1µg of recombinant spike protein alone, and 1µg spike protein in formulations including LQ, LMQ (liposomal (LS) formulations of QS-21 and MPL+QS-21 respectively), SQ, and SMQ (OIW emulsions of QS-21 and MPL+QS-21 respectively). Spike-specific antibody titers in arbitrary units (AU) were evaluated on (A) day 28 and (B) day 42 post-prime immunization, quantifying total IgG (left), IgG1 (right; a marker of Th2 polarized immunity), and IgG2a (right; a marker of Th1 polarized immunity) (Box-whisker plots: centre line: median; bounds: 25th/75th percentile; whiskers; lowest/highest point). A relative ratio of humoral isotypes IgG2a, a Th1-associated marker, and IgG1, a Th2-associated marker, were evaluated on (C) day 28 and (D) day 42. Median responses per treatment group were presented on a flower plot, following a log10-scale outlining predominantly Th2 polarized immunity in mice immunized with spike-alone, and a more Th1-like response in liposomal, and stronger Th1 associated response in mice immunized with OIW formulations. (E) Longitudinal weight analysis shows n significant changes in weight after prime or boost immunization. Aggregates shown, all individual weight changes were within 5% of weight prior to prime or boost immunization. Statistical significance was evaluated by Shapiro-Wilk test for normality, followed by Kruskal-Wallis and individual Wilcoxon rank-sum tests comparing vaccine formulations to vehicle control (PBS) and to antigen-alone (Spike). N = 10 / group. (* $p < 0.05$, ** $p < 0.01$, *** $p < 0.001$, and **** $p < 0.0001$).
